# Reliability and validity of the Chinese version of the COVID-19 Phobia Scale

**DOI:** 10.1186/s40359-022-01013-1

**Published:** 2022-12-18

**Authors:** Yuntian Xie, Ibrahim Arpaci, Yahui Xiao, Fanfei Meng, Ruotong Xie

**Affiliations:** 1 Department of Applied Psychology, Changsha Normal University, Changsha, China; 2 Department of Software Engineering, Bandirma Onyedi Eylul University, Balikesir, Turkey

**Keywords:** Chinese, Coronaphobia, The COVID-19 Phobia Scale, Perceived social support

## Abstract

**Background:**

The COVID-19 pandemic has become a source of fear worldwide and has negative mental health effects on the general population. In 2022, the epidemic continues to be characterized by many points, widespread and frequent in China, and the situation is serious and complex. To provide an effective and scientific tool, the study validated the Chinese version of the COVID-19 Phobia Scale (C19P-SC).

**Methods:**

This study selected 1138 Chinese individuals (age ranged 13 to 80).

**Results:**

Cronbach’s alpha coefficient for the C19P-SC was 0.93 (the coefficients of the four dimensions ranged from 0.75 to 0.85). The results of the confirmatory factor analysis supported the four-factor structure of the C19P-SC. Meanwhile, there was a positive and significant correlation between coronaphobia and state anxiety (*r* = 0.48, *p* < 0.001). The metric invariance hypothesis and the scalar invariance hypothesis were valid in the different subgroups. Significant multivariate effects of gender, education level, and identity differences on coronaphobia were found.

**Conclusions:**

The Chinese version of the COVID-19 Phobia Scale has good psychometric properties and is suitable for measuring COVID-19 phobia in Chinese individuals.

## Introduction

The COVID-19 pandemic has become a source of fear worldwide and has negative mental health effects on the general population [1–3]. In 2022, the epidemic continues to be characterized by many points, widespread and frequent in China, and the situation is serious and complex [4]. The topic of the pandemic continues to receive attention. With the COVID-19 pandemic, psychological problems are on the rise globally [5].

A recent meta-analysis showed that the mean fear of COVID-19 was high around the world [6]. An individual’s fear can lead to critical social and public health problems [2]. Fear of COVID-19 was strongly related to anxiety, traumatic stress, distress and so on [7]. A study that analyzed 17,865 active Weibo users based on an online eco-recognition method revealed that people reported higher levels of emotional problems during the pandemic (e.g., anxiety and depression) and cognitive problems (e.g., low life satisfaction and cognitive biases) [8]. Various emotional states, such as intolerance to ambiguity, psychological fragility, susceptibility to illness, and excessive anxiety during the pandemic, may manifest as coronaphobia [9].

Phobias are widely known as specific forms of anxiety. Phobias are manifestations of excessive and persistent fear of an entity or situation. Although coronaphobia, persistent and excessive fear of the novel coronavirus, is a specific phobia, it can be categorized as a particular type of the “DSM-V specific phobia” [10]. Recently, researchers have proposed a comprehensive model for understanding fear experiences during the COVID-19 pandemic [11]. According to this model, fearful experiences during the pandemic are categorized at the psychological level around four interrelated dialectical domains, namely, “(1) fear of the body/fear for the body; (2) fear of significant others/fear for significant others; (3) fear of not knowing/fear of knowing; (4) fear of taking action/fear of inaction” [11]. The “fear of COVID-19 scale” (FCV-19S) focuses mainly on two aspects (i.e., fear thinking and physical response to fear) [12]. The “COVID-19-Related Psychological Distress Scale” (CORPDS) focuses on two factor components (fear/anxiety and suspicion) [13].

Furthermore, the COVID-19 Phobia Scale (C19P-S) was developed using specific DSM-V phobia criteria. It has focused on four main factors: psychological, economic, somatic, and social [10]. Of these, the psychological factors focus primarily on fear and anxiety. Somatic factors are associated with perceived somatic symptoms. Economic factors refer to the hoarding of goods, and social factors mainly involve social anxiety. Individuals at higher risk of contracting COVID-19 are more likely to practice social distancing behaviors [14]. Changes in social interactions during the pandemic are complex phenomena involving health beliefs and demographic characteristics. These factors can define individuals with reduced social interaction early on and then perceive low social support [15].

At present, the C19P-S has been developed and adapted to multiple languages and cultures, including Turkish, English, Russian, Korean, and Japanese. However, a study with a Chinese population as subjects did not report the results of the reliability and validity tests of the COVID-19 Phobia Scale” [16]. They only reported the psychological and social dimensions when they applied the “COVID-19 Phobia Scale” to the Chinese samples. In addition, the sample source for this study was very limited (the samples selected were all from Wuhan, China). Therefore, the psychological properties of the Chinese version of the “COVID-19 Phobia Scale” still need to be analyzed and validated.

In summary, the present study aims to collect samples of various ages, educational levels, and occupations from different regions of China to investigate the psychological characteristics of the “COVID-19 Phobia Scale” in the Chinese population. Thus, this study will provide an effective and scientific tool to study COVID-19 phobia among the Chinese population. Because fear is associated with anxiety [7], some studies of the psychometric properties of this scale have used anxiety as a validated variable [10, 17], so this study will also test the anxiety level of the subjects. Furthermore, according to ecosystem theory, human development is greatly influenced by the mutual interactions between individuals and their actual social environments [18]. The presence of a significant other is assumed to provide supportive behaviors to a stressed person, thereby increasing the individual’s coping skills and minimizing stress-related symptoms [19]. An informed and intentional focus on social support is a noble endeavor that may benefit not just the youth but ultimately the society [20]. Social support can be classified as received social support and perceived social support [21]. Individuals’ subjective assessment of friend or family support during times of need is referred to as perceived social support. Researchers have found that perceived social support can weaken negative psychological symptoms such as anxiety, depression, and hopelessness [1, 22, 23]. Therefore, this study will also examine perceived social support related to coronaphobia during the COVID-19 pandemic to provide an empirical basis for effectively guiding people to cope with coronaphobia.

## Methods

### Participants

Questionnaires were distributed through an online surveying system named “Questionnaire Star” and 1138 valid and complete questionnaires were returned. Among them, 441 were males, and 697 were females. The mean age of the subjects was 25.48 (*SD* = 9.26, age ranged 13 to 80). The characteristics of the samples are shown in Table 1.

**Table 1:** Descriptive statistics of the participants

	*n*	%	Age (*M* ± *SD*)
1. Gender			
Male	441	38.75	26.44 ± 9.69
Female	697	61.25	24.88 ± 8.93
2. Educational level			
High school and below	133	11.69	37.45 ± 14.22
Junior college	129	11.34	26.94 ± 9.68
Undergraduate	761	66.87	22.81 ± 6.41
Postgraduate	115	10.11	27.55 ± 4.13
3. Identity			
Students	651	57.21	19.68 ± 2.61
Teachers	108	9.49	33.10 ± 8.47
Medical staff	161	14.15	28.72 ± 5.19
Retirees	11	0.97	57.50 ± 12.80
Others	207	18.19	34.96 ± 10.28
4. Chronic disease			
Yes	48	4.22	31.77 ± 15.31
No	1090	95.78	25.21 ± 8.81

### Measures

#### The COVID-19 Phobia Scale

We used the “COVID-19 Phobia Scale” (C19P-S) proposed by Arpaci et al. (2020) [9] in the study. The scale includes 20 items rated on a five-point Likert format. It consists of four factors, namely, psychological, somatic, social, and economic. A higher score indicates a more severe phobia. We developed a Chinese version of the scale (i.e., C19P-SC) in the current study. Initially, an assistant professor of psychology with a postgraduate degree translated the scale from English to Chinese. Then, an associate professor (Ph.D. in psychology) reviewed the content of the scale and made suggestions for changes. Following this, the scale was translated back into English. Cronbach’s *α* coefficient of the total scale in the current study was 0.93.

#### State-Trait Anxiety Inventory

The “State Anxiety Inventory” subscale of the State-Trait Anxiety Inventory (STAI) was proposed by Spielberger et al. [24] and adapted into Chinese by Zheng et al. (1993) [25]. The scale includes 20 items rated on a four-point Likert format. A higher score indicates more severe anxiety. This study calculated an overall Cronbach’s *α* coefficient of 0.88.

#### Perceived Social Support Scale

The “Perceived Social Support Scale” (PSSS) was developed by Zimet et al. (1988) [26] and adapted by Wang et al. (1999) [27]. The scale includes 12 items rated on a seven-point Likert format. It consists of three factors: friend support, family support, and other support. A higher score shows a higher level of perceived social support. The overall Cronbach’s *α* coefficient in the present study was 0.95.

### Procedure

Snowball sampling was used, and the questionnaire was distributed on the Internet (“Questionnaire Star”). Subjects were from the Hunan, Jiangxi, Jiangsu, Yunnan, Guangdong, and Hubei regions of China. All participants volunteered and received no compensation for their participation. Participants were not allowed to submit the questionnaire until they answered all questions.

### Data analysis

After organizing the data, SPSS 25.0 was utilized to perform correlation analysis, descriptive statistics, and difference testing. MPLUS 8.0 was used for confirmatory factor analysis and measurement invariance test. JASP0.16.1 was used to draw raincloud plots.

## Results

### Reliability

Cronbach’s alpha coefficient for the C19P-SC was 0.93. Adequate internal reliability consistencies (from 0.75 to 0.85) were calculated from the C19P-SC dimensions. Furthermore, normality was tested by using kurtosis and skewness coefficients. The findings demonstrated that the data were normally distributed. Descriptive statistics of the total scale and dimensions, kurtosis, skewness, and alpha values are provided in Table 2.

**Table 2:** Descriptive statistics, normality and reliability results

Dimension	*M* ± *SD*	Skewness	Kurtosis	*α*
Psychological	2.73 ± 0.81	0.01	−0.20	0.84
Somatic	1.79 ± 0.65	1.19	3.01	0.85
Social	2.45 ± 0.73	0.31	0.25	0.75
Economic	2.33 ± 0.79	0.37	0.05	0.80

### Convergent and discriminant validity

The “average variance extracted” (AVE) and “composite reliability” (CR) were calculated to test discriminant and convergent validity. If the CR was greater than 0.60, convergent validity could be accepted for the factors with an AVE less than 0.50 [10, 28, 29]. Table 3 shows the discriminant and convergent validity results.

**Table 3:** Convergent and discriminant validity

	CR	AVE	1	2	3	4	5
1.Psychological	0.84	0.48	1				
2.Somatic	0.84	0.53	0.57***	1			
3.Social	0.76	0.38	0.75***	0.69***	1		
4.Economic	0.79	0.49	0.74***	0.66***	0.75***	1	
5.Coronaphobia	0.93	0.41	0.90***	0.81***	0.91***	0.89***	1
6.State anxiety	0.80	0.25	0.41***	0.44***	0.43***	0.41***	0.48***

***p < 0.001

Four factors of the C19P-SC showed a positive and significant correlation with the total scores of coronaphobia and state anxiety. Furthermore, there was a positive and significant correlation between coronaphobia and state anxiety (*p* < 0.001). The result suggested that an individual with a higher level of coronaphobia also tends to exhibit a higher level of state anxiety. The overlap variability between the two structures was approximately 23.04%.

### Structural validity

The CFA results shown in Table 4 showed that the one-factor model fit poorly and the four-factor model fit better.

**Table 4:** Model fit indices for the measurement model

		*χ*^2^	*df*	χ^2^/*df*	CFI	TLI	RMSEA	SRMR
Model 1	1-Factor Model	2126.27	170	12.51	0.787	0.762	0.101	0.074
Model 2	4-Factor Model	828.81	154	5.38	0.927	0.909	0.062	0.055

### Measurement invariance test

Following the recommendation of Cheng and Rensvold (2002) [30], CFI ≤ 0.01 and RMSEA ≤ 0.015 was used as the criterion for measuring invariance in this study. The results showed (see Table 5) that the metric invariance hypothesis and the scalar invariance hypothesis were valid in the gender subgroup. Additionally, in the educational level subgroup, the metric invariance hypothesis and the scalar invariance hypothesis were valid. In the identity subgroup, because of the small sample size of retirees (*n* = 11), this study combined this group into the group named “others”. The results showed that the metric invariance hypothesis and the scalar invariance hypothesis were valid in the identity subgroup. Furthermore, because of the small sample size of those with chronic diseases (*n* = 48), no test of measurement invariance was performed for the subgroup with or without chronic diseases.

**Table 5:** Measurement invariance test

Model	χ^2^	*df*	CFI	RESEA [90% CI]	ΔCFI	ΔRMSEA
Gender						
M0 (configural)	1248.25	308	0.922	0.074 [0.069, 0.078]		
M1(metric)	1262.77	324	0.922	0.072 [0.067, 0.076]	0.000	0.002
M2 (scalar)	1327.44	340	0.918	0.072 [0.068, 0.076]	0.004	0.000
Educational level						
M0 (configural)	1772.54	616	0.905	0.082 [0.077, 0.086]		
M1(metric)	1819.68	664	0.906	0.078 [0.074, 0.083]	0.001	0.004
M2 (scalar)	1894.66	712	0.903	0.077 [0.073, 0.081]	0.003	0.001
Identity						
M0 (configural)	1795.77	616	0.904	0.082 [0.078, 0.087]		
M1(metric)	1843.49	664	0.904	0.079 [0.075, 0.084]	0.000	0.003
M2 (scalar)	1966.73	712	0.898	0.079 [0.075, 0.083]	0.006	0.000

### Group comparisons

**Fig. 1:**
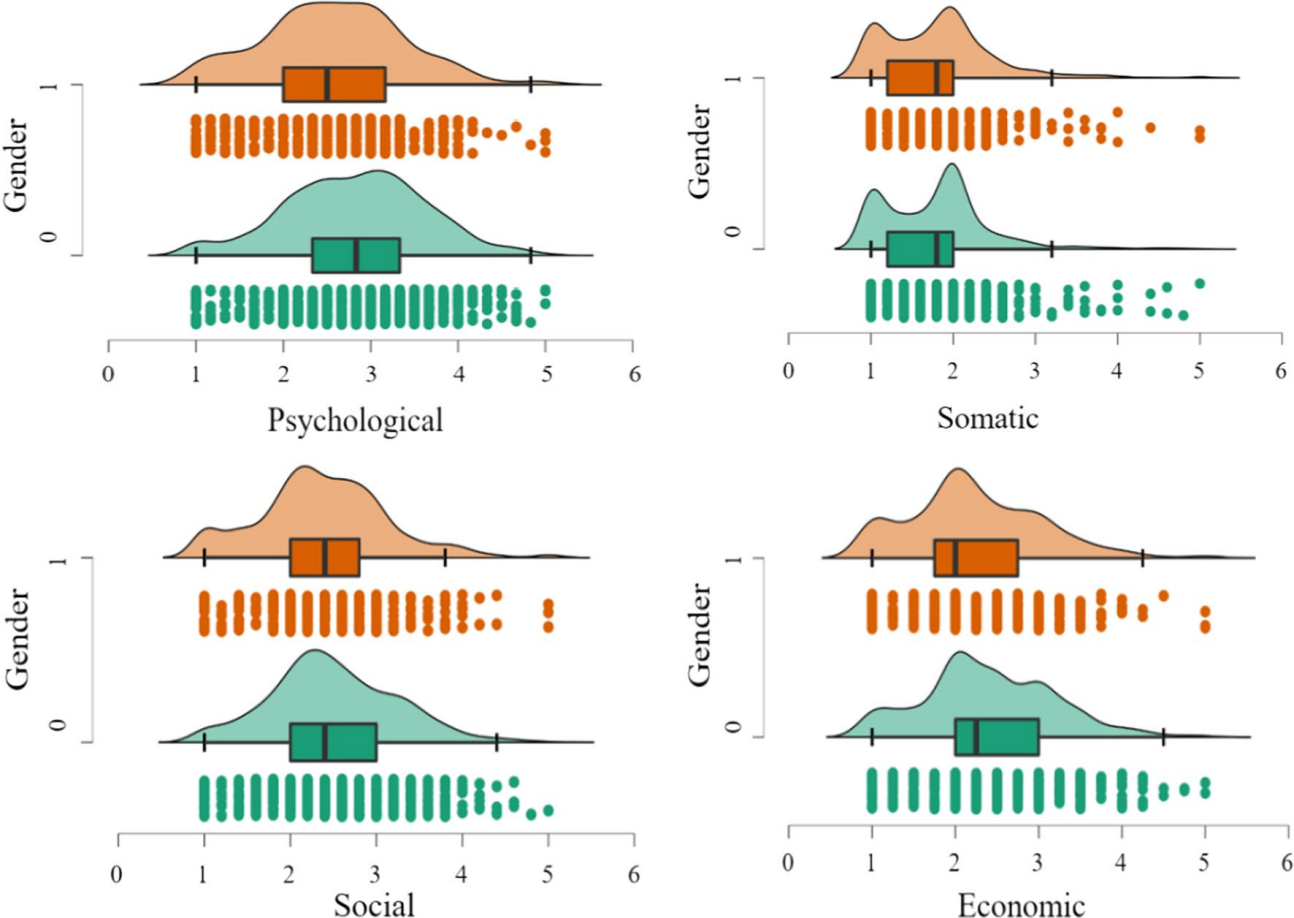
Raincloud plots of gender differences in coronaphobia*. Note* 1 = male, 0 = female

The results of two independent sample *t* tests showed that the gender difference in coronaphobia was significant, and the fear level of women (47.97 ± 12.75) was significantly higher than that of men (45.20 ± 13.39), *t* = 3.502, *p* < 0.001, *d* = 0.21. Specifically, females scored significantly higher than males on the psychological (*t* = 5.33, *p* < 0.001, *d* = 0.32), social (*t* = 2.91, *p* < 0.01, *d* = 0.18), and economic factors (*t* = 3.54, *p* < 0.001, *d* = 0.21). However, the gender differences in the somatic factor were not significant (*p* = 0.619) (see Fig. 1).

**Fig. 2:**
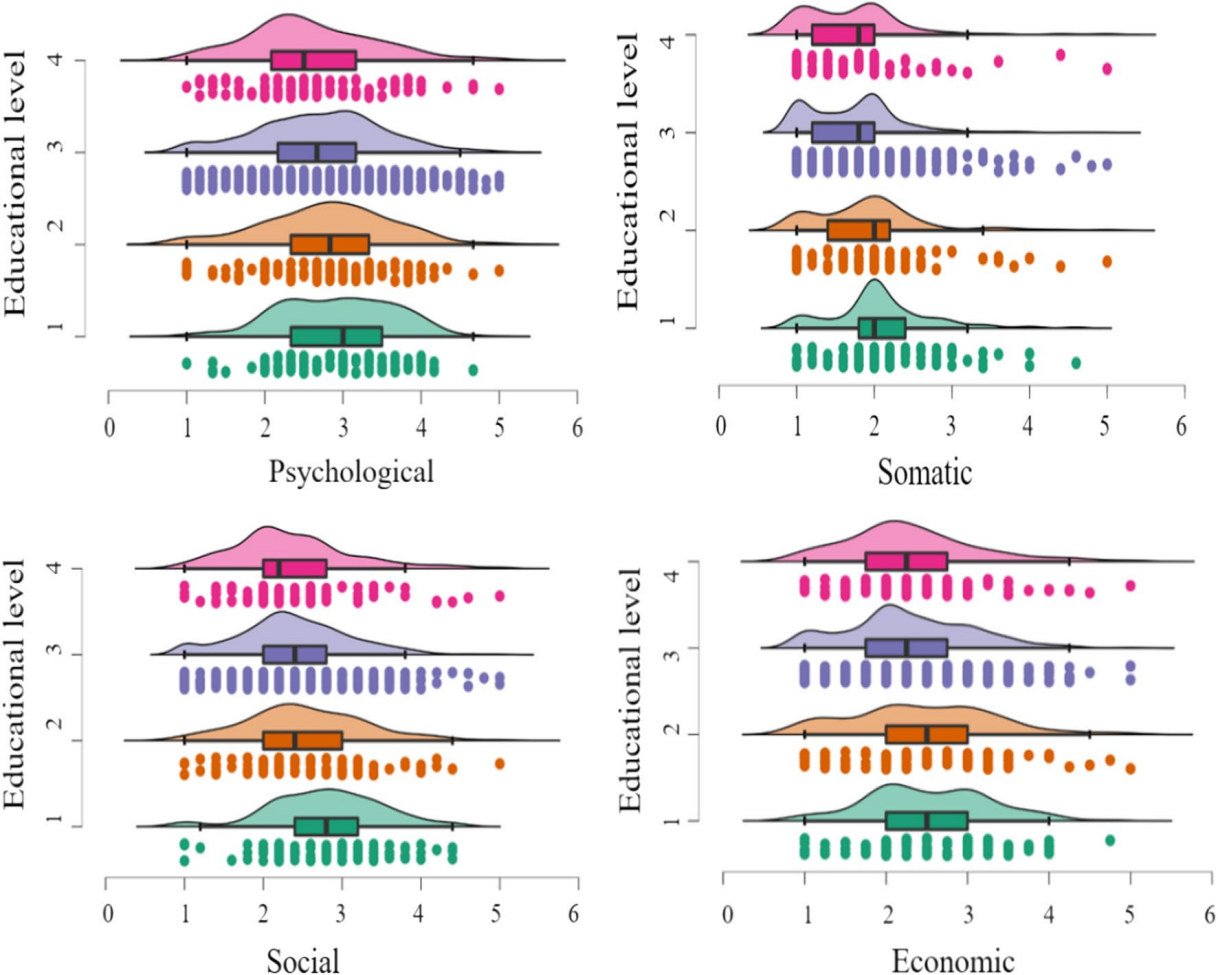
Rain cloud plots of educational level differences in coronaphobia*. Note* 1 = High school and below, 2 = Junior college, 3 = Undergraduate, 4 = Postgraduate

In addition, the results of one-way multivariate analysis of variance indicated a significant difference in education level in coronaphobia (*F* = 10.44, *p* < 0.001, *η*_*p*_^2^ = 0.03), the psychological factor (*F* = 4.32, *p* < 0.01, *η*_*p*_^2^ = 0.01), the somatic factor (*F* = 14.50, *p* < 0.001, *η*_*p*_^2^ = 0.04), the social factor (*F* = 13.89, *p* < 0.001, *η*_*p*_^2^ = 0.04), and the economic factor (*F* = 4.80, *p* = 0.003, *η*_*p*_^2^ = 0.013). Post hoc comparison results showed that individuals with high school and below levels scored significantly higher than those with undergraduate levels (*p* < 0.01) and postgraduate levels (*p* < 0.01) on the psychological factor, and individuals with junior college levels scored significantly higher than those with postgraduate levels (*p* < 0.05). Regarding the somatic factor, individuals with high school and below levels scored significantly higher, and individuals with junior college levels scored significantly higher than those with undergraduate levels (*p* < 0.01) and postgraduate levels (*p* < 0.05). Regarding the social factor, individuals with high school and below levels scored significantly higher than those with undergraduate levels (*p* < 0.01) and postgraduate levels (*p* < 0.05), and individuals with junior college levels scored significantly higher than those with undergraduate levels (*p* < 0.05). Regarding the economic factor, individuals with high school and below levels scored significantly higher, and individuals with junior college levels scored significantly higher than those with undergraduate levels (*p* < 0.05) (see Fig. 2).

**Fig. 3:**
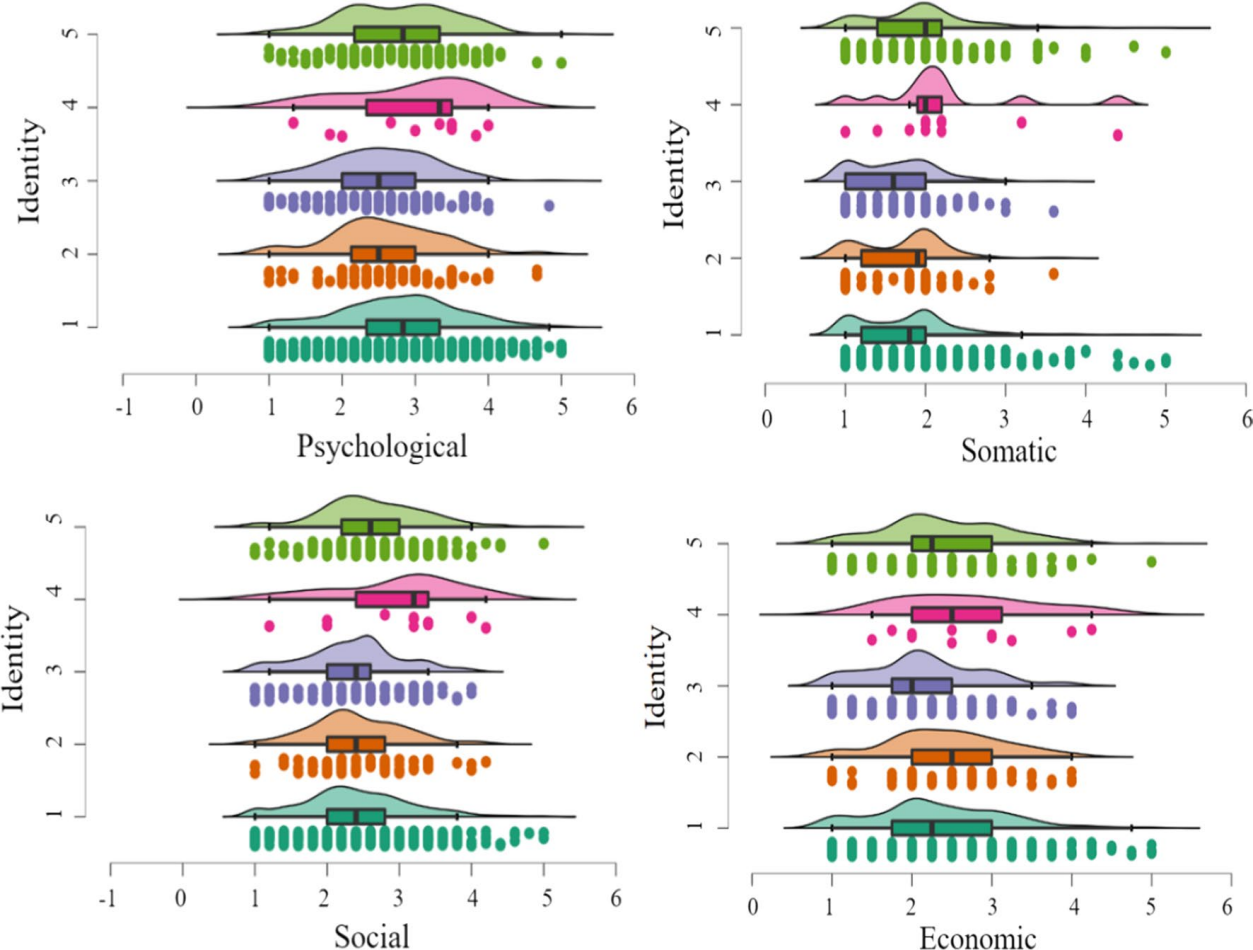
Rain cloud plots of identity differences in coronaphobia*. Note* 1 = Students, 2 = Teachers, 3 = Medical staff, 4 = Retirees, 5 = Others

Third, the results of one-way multivariate analysis of variance showed significant differences in terms of identity in coronaphobia (*F* = 5.39, *p* < 0.001, *η*_*p*_^2^ = 0.02), the psychological factor (*F* = 5.39, *p* < 0.001, *η*_*p*_^2^ = 0.02), the somatic factor (*F* = 7.22, *p* < 0.001, *η*_*p*_^2^ = 0.03), the social factor (*F* = 5.73, *p* < 0.001, *η*_*p*_^2^ = 0.02), and the economic factor (*F* = 3.41, *p* < 0.01, *η*_*p*_^2^ = 0.01). Post hoc comparison results showed that students scored significantly higher than teachers (*p* < 0.05) and medical staff (*p* < 0.001) on psychological factors, and other staff scored significantly higher than teachers (*p* < 0.05) and medical staff (*p* < 0.01). Regarding the somatic factor, students scored significantly higher than medical staff (*p* < 0.01); retirees scored significantly higher than students (*p* < 0.01), teachers (*p* < 0.05), and medical staff (*p* < 0.01). Regarding the social factor, retirees scored significantly higher than students (*p* < 0.05), teachers (*p* < 0.05), and medical staff (*p* < 0.01). Regarding the economic factor, medical staff scored significantly lowest (see Fig. 3).

Fourth, the two independent samples’ t test results did not show a significant difference in total score and four dimensions of coronaphobia based on chronic disease status. Furthermore, a linear regression analysis was conducted with perceived social support as the independent variable, related demographic variables as the control variable, and coronaphobia as the dependent variable. The results revealed that perceived social support significantly negatively predicted coronaphobia, *F* = 5.24***, *R*^2^ = 0.05, *β* = −0.06, *t* = −2.03, *p* < 0.05. Furthermore, the results shown in Table 6 indicated that perceived social support negatively and significantly predicted the somatic factor (*p* < 0.001) and economic factor (*p* < 0.05) but did not significantly predict the psychological factor (*p* = 0.97) or social factor (*p* = 0.12).

**Table 6:** The effect of perceived social support on coronaphobia

	Psychological		Somatic		Social		Economic	
	*β*	*t*	*β*	*t*	*β*	*t*	*β*	*t*
Male	−0.15	−4.96***	0.01	0.30	−0.09	−2.92**	−0.11	−3.67***
Age	−0.05	−0.96	−0.01	−0.19	0.02	0.46	0.01	0.04
High school and below	0.13	2.67**	0.16	3.49***	0.15	3.25**	0.08	1.69
Junior college	0.06	1.51	0.09	2.10*	0.05	1.29	0.05	1.14
Undergraduate	0.02	0.42	0.01	0.22	0.01	0.01	−0.02	−0.34
Students	0.05	0.81	0.01	0.15	−0.01	−0.21	0.02	0.38
Teachers	−0.02	−0.57	−0.02	−0.46	−0.01	−0.26	0.05	1.22
Medical staff	−0.07	−1.70	−0.07	−1.72	−0.04	−1.01	−0.05	−1.28
Retirees	0.03	0.85	0.03	1.08	0.04	1.16	0.03	0.82
Chronic disease	0.03	0.60	−0.01	−0.31	0.02	−0.77	0.03	1.08
Perceived social support	0.01	0.04	−0.13	−4.25***	−0.05	−1.56	−0.07	−2.30*
*F*	5.56***		6.57***		5.06***		3.86***	
*R*^2^	0.05		0.06		0.05		0.04	

*p < 0.05; **p < 0.01; ***p < 0.001

## Discussion

A large number of psychometric instruments have been developed for diagnosing, screening, or measuring COVID-19-related mental health problems since the emergence of the COVID-19 pandemic [3]. This study explored the psychometric characteristics of the Chinese “COVID-19 Phobia Scale” (C19P-SC), providing an effective measurement tool for screening COVID-19 phobia in the Chinese population.

The study found that both the total scale and the subscales have adequate internal reliability consistency. Furthermore, the CFA results and the convergent validity provide strong evidence for using the C19P-SC to assess the presence of coronaphobia in China. These results were consistent with prior research findings [10, 29, 31]. The C19P-SC is a measurement tool consisting of 20 items and includes four factors: psychological, somatic, social, and economic. Each factor has its significance and is related to each other and together constitutes the C19P-SC.

The study also compared groups based on demographic variables such as gender and education level. First, the results indicated that women had significantly higher levels of coronaphobia than men. Additionally, existing research suggests that women exhibit broader fears [32]. The gender difference is significant in psychological, economic, and social factors but not somatic factors. While Arpaci et al. (2022) [28] found that women experienced higher levels of fear only psychologically, no gender differences were found in other areas. This may be due to cultural influences or the origin of the subjects.

Furthermore, in terms of educational level, individuals with lower educational levels showed higher levels of coronaphobia. The study also points out that as education levels increase, individual coronaphobia levels decline. The scores on the four dimensions of the C19P-SC are higher for individuals at the high school level and below than those at other educational levels. The assumption is that individuals with a lower education level have less knowledge and a single source of information. Therefore, it is more challenging for them to approach the epidemic from a scientific, rational standpoint, and they are more likely to demonstrate more fear. Therefore, this study recommends that scientific knowledge on epidemic prevention and control be increased in the future. Furthermore, we should focus on traditional media, social media, and interpersonal communication to improve media literacy [33].

In addition, retirees and students showed higher levels of coronavirus fear in terms of identity. Retirees tend to be older and belong to the elderly group. They must come to terms with the end of their lives. Moreover, their access to information is relatively easy, and their connection with society has diminished. Existing studies have also found that the elderly have increased fear and loneliness during the COVID-19 pandemic [34].

Compared to elderly individuals, students are a young and active group. Their outlook on life and world outlook is still in development. A study reported that students were depressed, exhausted, nervous and angry due to COVID-19 [35]. School closings and home study due to an outbreak, combined with the lack of psychological support or counseling, would lead to intense negative emotions such as fear, anxiety, and depression. Therefore, teachers should pay close attention to the psychological changes of each student and guide them to release their emotions actively and maintain a healthy mind. In addition, this study also found that healthcare workers scored lower on coronaphobia in all four dimensions. Although fear is associated with a greater risk of exposure [36], healthcare workers are likely to be in close contact with infected or suspected cases. Nevertheless, because healthcare workers in China have adequate health protection, their levels of coronaphobia are low. Compared with students, teachers, etc., they know more about medical and health knowledge, including mental health knowledge, and do not exhibit apparent phobias.

An important finding from this study was that even after controlling for the effects of gender, age, and other factors, perceived social support could significantly impact an individual’s level of phobia. This speaks volumes about the protective role of the perceived social support. As perceived levels of social support increased, an individual’s coronaphobia levels decreased. An existing study also found that individuals who perceived more social support reported lower anxiety associated with COVID-19 [37]. From the perspective of the four dimensions of the C19P-SC, comprehension of social support mainly affects somatic and economic factors but has little effect on psychological and social factors. Because the consequences triggered by coronavirus infection are very serious [7], the psychological factor of this scale mainly refers to excessive anxiety and fear, and the social factor mainly assesses the degree of social fear [10], it is difficult to provide significant relief with only comprehending social support. In response to perceived social support, people will experience fewer somatic symptoms caused by psychological fear. This suggests that individuals who experience physical symptoms should receive more social support and lessen the negative psychological impact on the body. In addition, if a sufficient supply of goods can be guaranteed and the public can fully perceive this, the hoarding behavior caused by coronaphobia will be considerably alleviated.

This study also has some limitations. For example, the Chinese subjects selected in this study are predominantly from southern China (including southeast, southwest, and south-central) and fewer from northern areas. Future research can select subjects from a wider area and explore them in more depth. A second limitation of this study is that no distinction was made between clinical samples, especially those with phobias, and nonclinical samples. Future research can provide a more detailed analysis of this issue. In addition, there was a relatively high correlation between the four dimensions of the C19P-SC. Therefore, the discriminant validity of the scale should be judged with caution. This hypothesis needs to be further validated by future studies.

## Conclusion

The Chinese version of the “COVID-19 Phobia Scale” (C19P-SC) has good psychometric properties and is suitable for measuring COVID-19 phobia in the Chinese population. Furthermore, improving the perception of social support can decrease coronaphobia in people.

## Data Availability

The datasets used and/or analysed during the current study available from the corresponding author on reasonable request.
